# A long-term "memory" of HIF induction in response to chronic mild decreased oxygen after oxygen normalization

**DOI:** 10.1186/1471-2261-7-4

**Published:** 2007-01-18

**Authors:** Chandrashekhar D Kamat, Jessica E Thorpe, Satyendra S Shenoy, Antonio Ceriello, Dixy E Green, Linda A Warnke, Michael A Ihnat

**Affiliations:** 1 Department of Cell Biology, University of Oklahoma Health Sciences Center, Oklahoma City, OK 73104, USA; 2 Warwick Medical School, University of Warwick, Gibbett Hill, Coventry CV4 7AL U.K; 3 Department of Pharmacology, Nicholas Piramal Research Centre, Mumbai, India

## Abstract

**Background:**

Endothelial dysfunction (ED) is functionally characterized by decreased vasorelaxation, increased thrombosis, increased inflammation, and altered angiogenic potential, has been intimately associated with the progression and severity of cardiovascular disease. Patients with compromised cardiac function oftentimes have a state of chronic mild decreased oxygen at the level of the vasculature and organs, which has been shown to exacerbate ED. Hypoxia inducible factor (HIF) is a transcription factor complex shown to be the master regulator of the cellular response to decreased oxygen levels and many HIF target genes have been shown to be associated with ED.

**Methods:**

Human endothelial and aortic smooth muscle cells were exposed either to A) normoxia (21% O_2_) for three weeks, or to B) mild decreased oxygen (15% O_2_) for three weeks to mimic blood oxygen levels in patients with heart failure, or to C) mild decreased oxygen for two weeks followed by one week of normoxia ("memory" treatment). Levels of HIF signaling genes (HIF-1α, HIF-2α, VEGF, BNIP3, GLUT-1, PAI-1 and iNOS) were measured both at the protein and mRNA levels.

**Results:**

It was found that chronic exposure to mild decreased oxygen resulted in significantly increased HIF signaling. There was also a "memory" of HIF-1α and HIF target gene induction when oxygen levels were normalized for one week, and this "memory" could be interrupted by adding a small molecule HIF inhibitor to the last week of normalized oxygen. Finally, levels of ubiquitylated HIF-1α were reduced in response to chronic mild decreased oxygen and were not full restored after oxygen normalization.

**Conclusion:**

These data suggest that HIF signaling may be contributing to the pathogenesis of endothelial dysfunction and that normalization of oxygen levels may not be enough to reduce vascular stress.

## Background

Endothelial dysfunction has been shown to play a causative role in atherosclerosis [1], ischemic heart disease [2], coronary artery disease [3], peripheral neuropathy [4], diabetic cardiomyopathy [5], erectile dysfunction [6] and even ischemia/reperfusion subsequent to myocardial infarction [7]. Endothelial dysfunction is a generalized syndrome characterized by attenuated vasodilation [8], due at least in part to decreased available nitric oxide (NO) [9], a concomitant buildup of reactive species [10] and stimulation of vascular stress pathways [11]. Another characteristic of endothelial dysfunction is an altered angiogenic response [12], which can affect tissue vascularity, immune cell infiltration, blood flow patterns, and wound healing (reviewed in [11]). If the blood vessels cannot supply the tissues with blood, a state of relative decreased oxygen ensues further contributing to endothelial dysfunction [13].

Hypoxia induces expression of hypoxia inducible factor (HIF), a transcription factor complex shown to be the master regulator of the cellular response to decreased oxygen levels [14]. The HIF proteins belong to a family of basic helix-loop-helix transcription factors. The HIF complex is a heterodimer consisting of a regulated α subunit and a constitutive β subunit [15]. In normoxic environments, the α subunit is hydroxylated at proline residues [16], which targets it for ubiquitylation, by proteins such as Von Hippel-Lindau [17], ultimately resulting in its degradation by the proteasome. There are two known HIF α subunits which stimulate transcription, HIF-1α and HIF-2α, in addition to a third α subunit (HIF-3α) lacking the DNA binding domain which appears to compete with HIF-1α and HIF-2α for β subunit binding and acts to inhibit HIF signaling [18]. The HIF-1α/HIF-1β heterodimer binds to hypoxia response elements in the regulatory regions of more than 70 target genes such as the vascular endothelial growth factor (VEGF), plasminogen activator inhibitor (PAI-1), inducible nitric oxide synthase (iNOS), erythropoietin, Bcl2/adenovirus EIB 19kD-interacting protein 3 (BNIP3), heme oxygenase-1, and glucose transporter-1 (GLUT-1) [19]. In addition to being regulated by hypoxia, the HIF pathway can be modulated by reactive species [20,21], nitric oxide [22], metals [23,24], cytokines [25], and growth factor signaling through phosphatidylinositol 3-kinase [26] and mitogen activated protein (MAP) kinase pathways [27].

The idea that vascular stress has long-lasting effects that perpetuate further damage, even when the stressor is removed, is developing support both in the laboratory [28] and in the clinic [[29-32] 364, 771-7]. For example, it was found that changes in cellular fatty acid metabolism persist for up to 30 hours after oxygen levels are normalized following myocardial ischemia [31]. Clinically, persistent benefits of antihypercholesterolemic agents [32] angiotensin converting enzyme (ACE) inhibitors [33], and insulin- and drug-mediated glucose normalization in diabetic patients [29,30] have been observed long after these agents were discontinued, further supporting a what has been termed a "memory" of vascular stress.

The first goal of this paper was to determine whether chronic exposure to mild decreased oxygen (15% O_2_) induces the HIF signaling system in blood vessels. The second goal was to examine whether there was a "memory" of HIF signaling induction long after oxygen levels were normalized. To investigate these novel questions, human endothelial and aortic smooth muscle cells were exposed either to A) normoxia (21% O_2_) for three weeks, or to B) mild decreased oxygen (15% O_2_) for three weeks, or to C) mild decreased oxygen for two weeks followed by one week of normoxia ("memory" treatment). Levels of HIF signaling genes (HIF-1α, HIF-2α, VEGF, BNIP3, GLUT-1, PAI-1 and iNOS) were measured at the protein and mRNA levels. It was found that chronic exposure to mild decreased oxygen resulted in significantly increased HIF signaling. There was also a "memory" of HIF-1α protein and of several HIF target genes when oxygen levels were normalized for one week after two weeks of mild decreased oxygen, and this "memory" could be interrupted by adding a chemical HIF inhibitor to the last week of normalized oxygen. Finally, levels of ubiquitylated HIF-1α were reduced in response to chronic mild decreased oxygen and were not full restored after oxygen normalization. Taken together, these data suggest a "memory" of HIF signaling through HIF-1α in the endothelium in response to chronic mild decreased oxygen and point out the need for more aggressive vascular drug therapies for overcoming the vascular "memory" stress response.

## Methods

### Chemicals and cell culture reagents

All chemicals were purchased from EMD Biosciences (San Diego, CA) unless otherwise noted. Cell culture reagents were purchased from Invitrogen (Carlsbad, CA). Inhibitors were purchased from EMD Bioscience except oxypurinol and ALA, which were from Sigma Chemical and the UCP2 adenovirus, which was a kind gift of Dr. Ming-Hui Zou in Dept. of Endocrinology at OUHSC.

### Cell culture

HMEC-1 human microvascular endothelial cells [34] (obtained from the Centers for Disease Control) were grown in MCDB-131 (pH 7.5 ± 0.3) media supplemented with 15% Cosmic Calf Serum (Hyclone, Logan, UT), 2 mM sodium pyruvate, 2 mM glutamine (Mediatech, Herndon, VA) and penicillin/streptomycin (Hyclone, Logan, UT), 10 ng/ml EGF (Peprotech, Rocky Hill, NJ), and 1 μg/ml hydrocortisone in a humidified environment with 5% carbon dioxide added. HUVEC (human umbilical vein endothelial cells) and ASMC (human aortic vascular smooth muscle cells) (Cambrex, North Brunswick, NJ) were grown in Media-199 (Mediatech, Herndon, VA) supplemented with 15% Cosmic Calf Serum (Hyclone, Logan, UT), 2 mM sodium pyruvate, 2 mM glutamine (Mediatech, Herndon, VA), penicillin/streptomycin (Hyclone, Logan, UT), 1% MEM Vitamins (Invitrogen Carlsbad, CA) and 1% MEM Non-essential Amino Acids (Invitrogen Carlsbad, CA) in a humidified environment with 5% carbon dioxide added. Cells were incubated with 5 mM glucose (with 25 mM mannitol for osmolarity normalization). Decreased oxygen levels were achieved using a 3-gas incubator (NuAire, Plymouth, MN) and cells fed and passaged in decreased oxygen using a hypoxic glove box (Coy, Ann Arbor, MI). As a control for HIF stimulation, cells were exposed for 4 hr to 2.5% O_2 _[35] or to 100 μM deferoxamine for 4 hr [36]. The last week of normoxia was given with or without 62.5 μM α-lipoic acid (ALA) [37], or with or without 10 μM apocynin [38], or with or without 1 mM L-NAME [39], or with or without 10 μM oxypurinol [39], or with or without 5 μM YC-1 [40], or with or without 25–100 plaque-forming units (PFU)/cell of an UCP2 overexpressing adenovirus [41]. Inhibitors were replaced every other day and 24 hours before the end of the experiment. Cells were grown at 25–30% confluence in 25 or 75 cm^2 ^flasks until 100% confluent.

### Adenoviral siRNA constructs

Sequences for the three HIF-1α small hairpin interfering RNAs are previously published [42-44]. Sequences were synthesized by the OUHSC molecular biology resource facility and ligated first into a shuttle vector (pShuttle-CMV, Stratagene, Cedar Creek TX) and then into an adenoviral vector (pAdEasy-1, Stratagene, Cedar Creek TX), cotransformed into competent bacterial cells containing adenoviral plasmid (BJ1583, Stratagene, Cedar Creek TX), linearized and transfected into HEK293 competent cells (Stratagene, Cedar Creek TX). Adenoviral titer was determined by a plaque agarose assay and 50–100 PFU/cells of each virus in serum-free media for 2 hr then serum-containing media replaced. Cells were re-treated every 3 days.

### CFDA-AM cell proliferation assay

After treatment, 5 μM 5-carboxyfluorescein diacetate acetoxymethyl ester (CFDA-AM; Molecular Probes, Eugene, OR), a substrate cleaved multiple times by non-specific cellular esterases to form fluorescein, was added for 1 h at 37°C. The fluorescence was read on a plate reader (BMG Labtech, Durham, NC) using the fluorescein channel as a marker of cell proliferation [45]. Data were then graphed as a percent of the fluorescence of untreated or normoxic cells.

### Co-immunoprecipitation and Western blot

Cell lysates (100 μg) were immunoprecipitated using an antibody against HIF-1α (2 μg/100 μg of sample protein) (anti-rabbit, Chemicon, Littleton, CO) and subjected to SDS-PAGE, transferred to nitrocellulose and immunoblotted for ubiquitin (Ubiquitin anti-rabbit 1:1500, Abcam, Cambrige, MA), and immmunoblotted against HIF-1α (anti-mouse, 1:100, Abcam H1alpha67, Cambrige, MA) used as a control for immunoprecipitation.

Equal amounts of cell lysates (20–50 μg) were resolved using 8–12% SDS-PAGE gels, transferred to 0.2 μm nitrocellulose (Schleicher and Schuell), blocked in SuperBlock (Pierce Chemical), incubated with primary antibodies overnight at 4°C or for 2 hours at room temperature, washed three times in Tris-buffered saline with 0.25% Tween-20 (TBST), appropriate secondary antibodies anti-mouse or anti-rabbit HRP-conjugates (Pierce) diluted in StartingBlock (TBS) Blocking Buffer added at 1:25,000 dilution for 1 hr at RT, washed three times with TBST, SuperSignal Dura chemiluminescence substrate (Pierce Chemical) added, and digital images captured using a charge-coupled device (CCD) camera (Kodak Image Station 4000). Primary antibodies and dilutions used were as follows: anti-HIF-1α(Chemicon: 1:500), anti-HIF-2α (Santa Cruz Biotechnology: 1:500), anti-Glut-1 (Santa Cruz Biotechnology: 1:150), anti-iNOS (Upstate Biotechnology, Lake Placid, NY: 1:500), anti-BNIP3 (Abgent, San Diego, CA 1:100), anti-PAI-1 (Santa Cruz Biotechnology: 1:150)). Densitometry of bands was done using Image J 1.30 analysis software capturing integrated density of regions of interest and dividing these values by the unit area of the region of interest. In all cases, confirmation of equal loading was accomplished by Memcode staining (Pierce Chemical) and by probing blots against vinculin (1:4,000 Sigma).

### RNA Extraction and Quantitative Real-Time RT-PCR

Total RNA was extracted from the control and treated HMEC-1 cells using the RNeasy kit (QIAGEN) immediately following the treatments. The concentration and purity of RNA samples were determined spectrophotometrically by absorbance at 260 and 280 nm, and the integrity was confirmed by agarose gel electrophoresis. Synthesis of the first strand cDNAs, quantitative real-time PCR reactions and data analysis were then performed. Total RNA (1 μg) was reverse transcribed in 50 μl reaction and 5 μl of cDNA was then used for PCR reaction according to Invitrogen technical manual.

The following gene-specific primer pairs were used: *hif-1α*, forward; 5'-CTGATATTAAACCTAAATGTTCTGCCTACC-3', reverse; 5'-CAGTCTGCTCAAAATAT CTTTATACCAAC-3'; *glut-1*, forward 5'-CCTAAGGATCTCTCAGGAGCACAG-3', reverse; 5'-TCAGGTTTGGAAGTCTCATCCAG-3'; *vegf*, forward; 5'-TGTATTTGAC TGCTGTGGACTTGAG-3', reverse; 5'-TCAGGATCTGAGTGGGAACATTC-3', *hif-2α*, forward 5'-GCGGCCGCTCACTTGTCATCGTCGTCCTTGTAGTCGGTGGCCTGGT-3', reverse; 5'-ACCAGGCCACCGACTACAAGGACGACGATGACAAGTGAGCGGCCGC-3', *iNOS*, forward, 5'- TACTCCACCAACAATGGCAA-3', reverse, 5'- GATGAGCTGAGCATTCCACA-3', *hprt, forward *5'-GGGAGGCCATCACATTGTAG-3', reverse, 5'-CCTGACCAAGGAAAGCAAAG-3'. Gene expression of HIF-1α, HIF-2α, iNOS, Glut-1, and VEGF were quantified relative to the expression level of HPRT, a housekeeping gene. Probes for these reactions were purchased from Invitrogen (Carlsbad, CA). The difference in threshold number of cycles between the gene of interest and HPRT was then calculated and plotted as relative abundance of gene of interest over HPRT.

### VEGF ELISA

VEGF levels were assessed by ELISA using a rabbit polyclonal anti-VEGF antibody (Abcam, Cambridge, MA). Supernatants from cells (100 μl) were added to a high binding white ELISA plate (COSTAR, Corning, NY) together with a standard curve of recombinant human VEGF (0.1 – 10,000 pg/ml) (Biosource International, Camarillo, CA) overnight at 4°C. The wells were washed with TBS, blocked with 1% BSA in TBS before anti-VEGF antibody was (1:1000 dilution of 1 mg/ml stock) added for 2 hours at RT, the wells washed again and 1:2000 anti-Rabbit HRP conjugate (Pierce, Rockford, IL) added for 45 minutes at RT, the wells washed again with TBS, then an ECL ELISA substrate (Pierce) added to wells for 5 minutes and the chemiluminescence measured using a plate reader (Fluostar Optima, BMG Labtechnologies, NC). Data were normalized to the standard curve to yield values in pg/ml VEGF and graphed as percentage of untreated control.

### Statistics

Data were analyzed using one-way analysis of variance to compare the means of all the groups. The Neuman-Keuls multiple comparisons procedure was used to determine which pairs of means were different. Differences were considered significant at *P *< 0.05.

## Results

### Chronic mild decreased oxygen induces the HIF pathway

**Figure 1:**
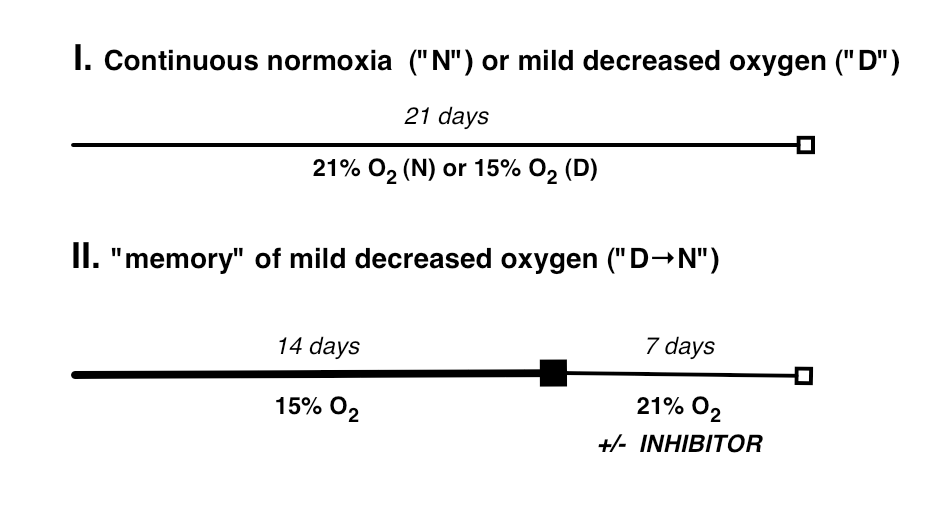
**Treatment scheme for experiments in this work**. Isolated microvascular endothelial cells mild decreased oxygen (15% O_2_) for three weeks (I) or to mild decreased oxygen for two weeks then to normoxia for one week ("memory" treatment – II).

**Figure 2:**
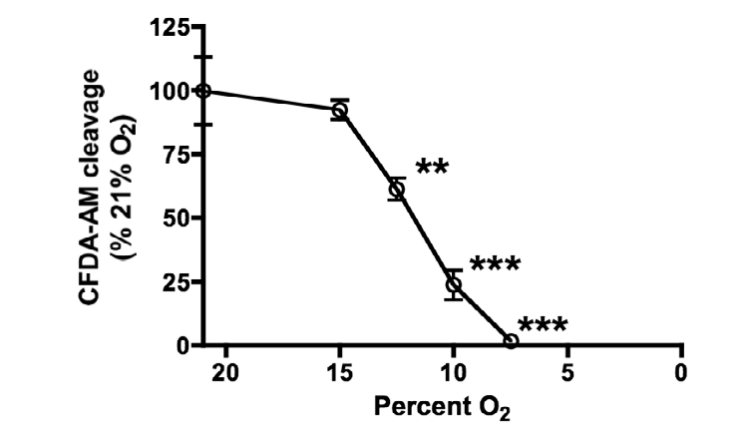
**Effect of chronic relative hypoxia on a marker of cell proliferation in HMEC-1 cells**. HMEC-1 cells were exposed to normoxia (20% O2) or to various levels of relative hypoxia for 3 weeks followed by a one hour incubation with 5 μM CFDA-AM and fluorescence measurement as in methods. The data represent the mean +/- SEM of triplicate wells from 3 independent experiments. ** = P < 0.01 and *** = P < 0.001 using one-way ANOVA with a Neuman-Keuls multiple comparisons post-test.

Human microvascular endothelial cells (HMEC-1) were exposed to either 21% O_2 _(normoxia) or to 5%, 7.5% and 10 and 15% O_2 _for three weeks (Figure 1). Oxygen levels lower than 15% O_2 _were found to result in significant decreases in endothelial cell proliferation (Figure 2). Since the goal of this paper was to examine the effect of *mild *decreased oxygen, 15% O_2 _was used for the remainder of the studies. This level of decreased oxygen is very mild in comparison with arterial oxygen levels of patients with chronic obstructive pulmonary disease [46,47], sleep apnea [48], or coronary artery disease [49].

**Figure 3:**
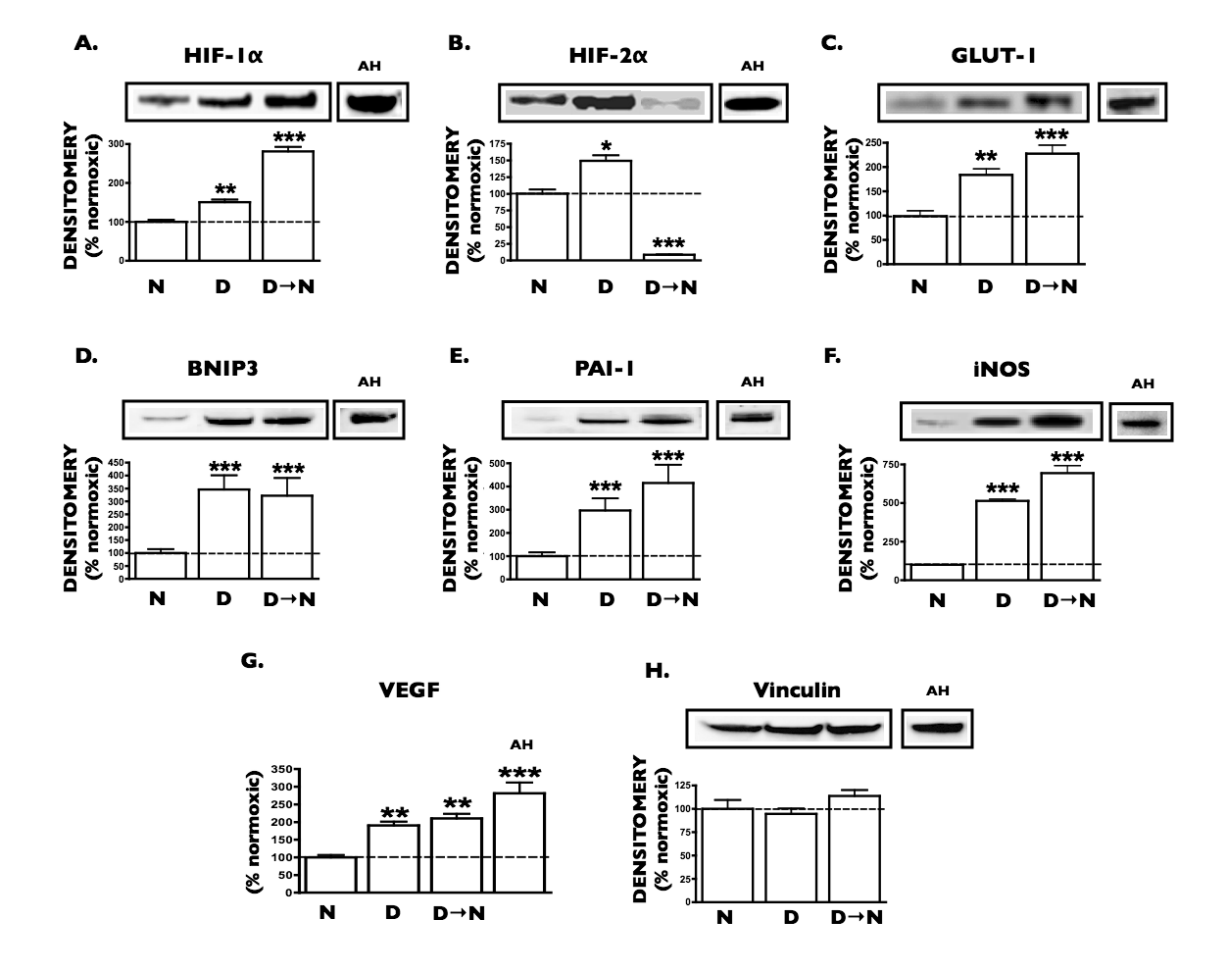
**Effect of oxygen normalization on HIF-1α, HIF-2α, Glut-1, BNIP3, PAI-1, iNOS and VEGF protein expression in human endothelial cells**. Human microvascular endothelial cells were exposed as described in Figure 1. Western blots were performed against HIF-1α (A), HIF-2α (B), Glut-1 (C), BNIP3 (D), PAI-1 (E), iNOS (F) on cellular lysates and ELISA was performed against VEGF (G) on cellular supernatants. Graphs indicate the densitometric analysis relative to the respective normoxic protein expression (dashed line) with a representative blot for each protein above each graph. Acute hypoxia (2.5%0_2_; 4 hours) ("AH") was used as a positive control for the induction of proteins. Data in graphs represent the mean +/- S.E.M. from three separate experiments with a total sample size of 4–6. Asterisk, *P *< 0.05; double asterisk, *P *< 0.01; triple asterisk, *P *< 0.001 as compared to cells incubated in normoxia for three weeks.

**Figure 4:**
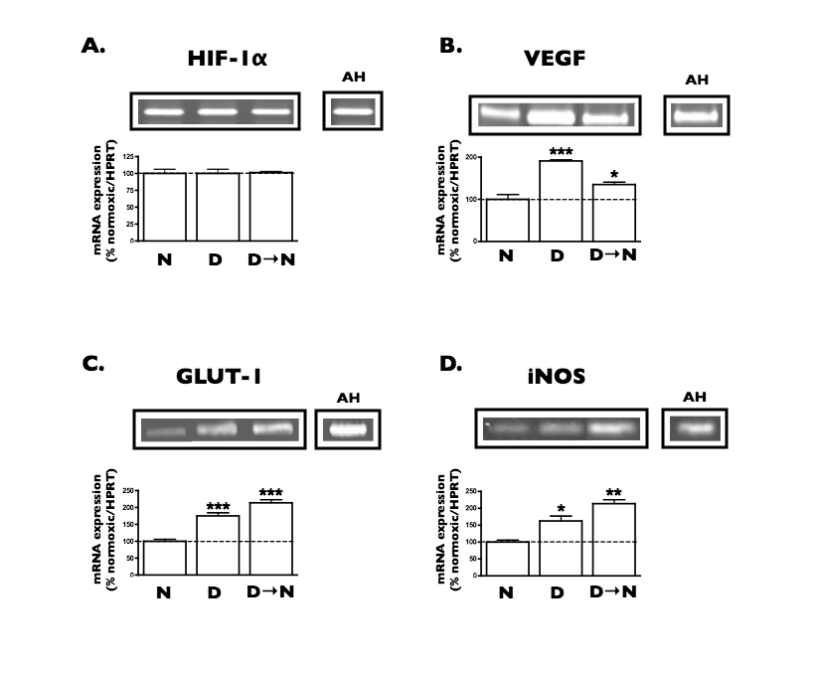
**Effect of oxygen normalization on HIF target mRNA expression in human endothelial cells**. Human microvascular endothelial cells were exposed as described in Figure 1. Total RNA was extracted and the amount of HIF-1α (A), VEGF (B), Glut-1 (C), and iNOS (D) transcripts were analyzed by quantitative real-time RT-PCR as described in Materials and Methods. In the graphs (bottom panels), all values are expressed relative to the HPRT gene expression for each condition. Acute hypoxia (2.5% 0_2_; 4 hours- "AH") was used as a positive control for the induction of transcripts. Data in graphs represent the mean +/- S.E.M. from three separate experiments with a sample size of 3–5 with representative gels above each graph. Asterisk, *P *< 0.05; double asterisk, *P *< 0.01; triple asterisk, *P *< 0.001 as compared to cells incubated in three weeks of normoxia (21%0_2_) (**N**) (dashed line).

Levels of HIF signaling proteins (Figure 3) and steady-state mRNA (Figure 4) were measured by Western blot and real-time RT-PCR, respectively. For each protein or mRNA graph, representative Western blots (for protein except for VEGF in which an ELISA was used) or RNA gels (for mRNA) are shown above graphs of densitometric analysis of bands. We found that protein levels of HIF-1α and HIF-2α and of the HIF target genes GLUT-1 [50], BNIP3 [51], PAI-1 [52], iNOS [53], and VEGF [54] were induced in response to chronic mild decreased oxygen (Figure 3A–G; "D" compared to "N"), although this induction was not as profound as that of the cells in response to acute hypoxia (2.5% O_2 _for 4 hours; Figure 3A–G; ("AH")). In contrast, little effect was observed on the constitutively regulated loading protein vinculin (Figure 3H). It was next found that there was no changes in HIF-1α (Figure 4A) mRNA levels in response to chronic mild decreased oxygen, but that mRNA levels of the HIF target genes VEGF (Figure 4B), GLUT-1 (Figure 4C), and iNOS (Figure 4D) were induced as compared to normoxic controls.

### HIF induction persists after oxygen normalization

To determine whether the induction of HIF signaling persists after oxygen levels are normalized, cells were exposed to two weeks of mild decreased oxygen followed by one week of normoxia. Unexpectedly, levels of HIF-1α protein (Figure 3A) but not HIF-1α mRNA (Figure 4A) remained induced one week after normalization. There was no "memory" of HIF-2α protein induction (Figure 3B) but there was a "memory" of induction of the HIF target genes GLUT-1 (Figure 3C), BNIP3 (Figure 3D), PAI-1 (Figure 3E), iNOS (Figure 3F) and VEGF (Figure 3G) at the protein level. This "memory" of induction was also observed for VEGF (Figure 4B), GLUT-1 (Figure 4C) and iNOS (Figure 4D) at the mRNA level.

### The "memory" of HIF signaling after oxygen normalization is not cell specific

**Figure 5:**
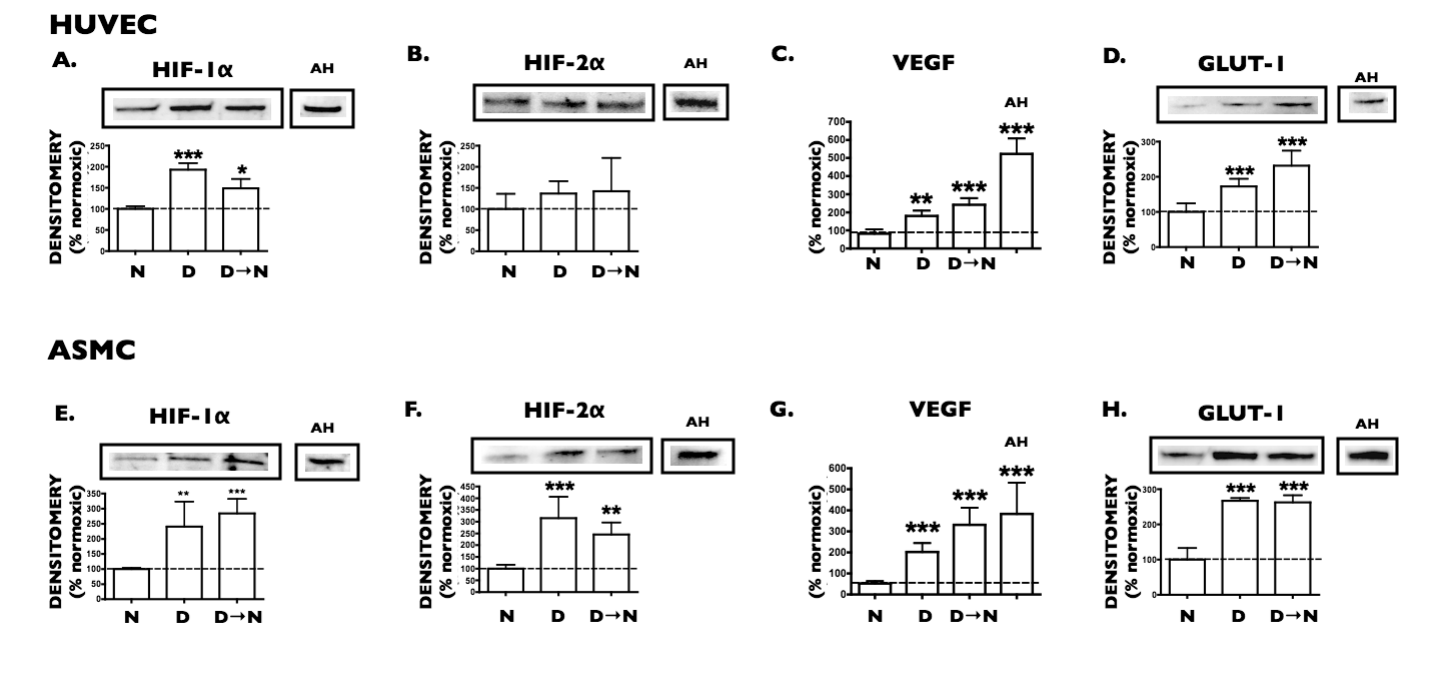
**Effect of oxygen normalization on expression of HIF-1α, HIF-2α, VEGF, and GLUT-1 protein expression in HUVECs and ASMCs**. HUVECs (A-D) and human ASMCs (E-H) were exposed as described in Figure 1. Western blots were performed against HIF-1α (A and E), HIF-2α (B and F), GLUT-1 (D-H) on cellular lysates and ELISA was performed against VEGF (C and G) on cellular supernatants. Graphs indicate the densitometric analysis relative to the respective normoxic protein expression (dashed line) with a representative blot for each protein above each graph. Acute hypoxia (2.5% 0_2_; 4 hours – "AH") was used as a positive control for the induction of each of the target protein. Data in graphs represent the mean +/- S.E.M. from three separate experiments with a total sample size of 4–6 and with representative gels above each graph. Asterisk, *P *< 0.05; double asterisk, *P *< 0.01; triple asterisk, *P *< 0.001 as compared to cells incubated in normoxia for three weeks.

To determine whether this "memory" of HIF induction was unique to the HMEC-1 cells, which harbor an SV40 T antigen [34], primary human umbilical vein endothelial cells (HUVECs) and human aortic vascular smooth muscle cells (ASMCs) were exposed to the same treatments as the HMEC-1 cells. Like the HMEC-1s, both an induction of HIF-1α (Figure 5A, 5E) and the HIF target genes VEGF (Figure 5C, 5G) and GLUT-1 (Figure 5D, 5H) and a "memory" of that induction was observed at the protein level in both the HUVECs and ASMCs. Interestingly, an absence of induction of HIF-2α was observed in the HUVECs (Figure 5B) but not in the ASMCs (Figure 5F).

### HIF mediates in the persistent induction of VEGF, BNIP3, PAI-1 and iNOS after oxygen normalization

**Figure 6:**
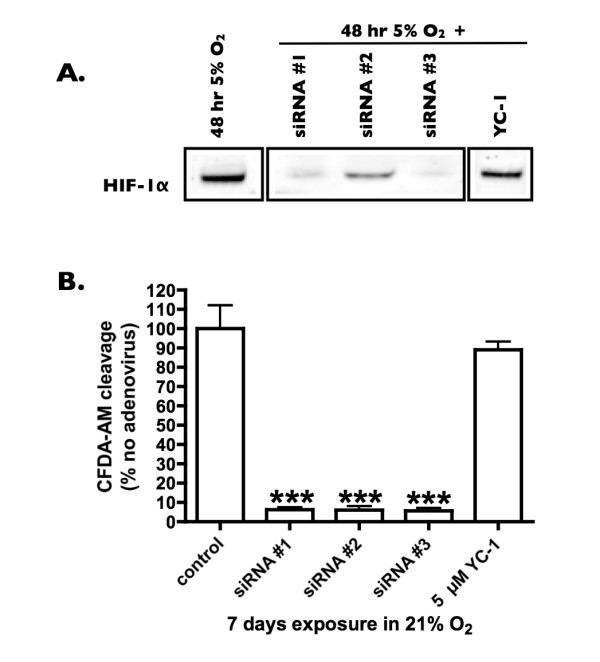
**Effect of adenoviral siRNA on HIF-1α protein (A) and cell proliferation (B) in HMEC-1 cells**. Adenoviral vectors containing hairpin siRNAs against HIF-1α were created as in the methods. Panel A. HMEC-1 cells were infected with 50–100 PFU/cell adenovirus or 5 μM YC-1, exposed to 5% O_2 _for 48 hours then cell lysates made and HIF-1α protein expression assessed by Western blot. Representative bands for HIF-1α are shown. Panel B. HMEC-1 cells were infected with adenovirus or treated with 5 μM YC-1 every 3 days for 7 days and cell proliferation assessed using the CFDA-AM assay as in methods. The data represent the mean +/- SEM of triplicate wells from 3 independent experiments. *** = P < 0.001 using one-way ANOVA with a Neuman-Keuls multiple comparisons post-test.

**Figure 7:**
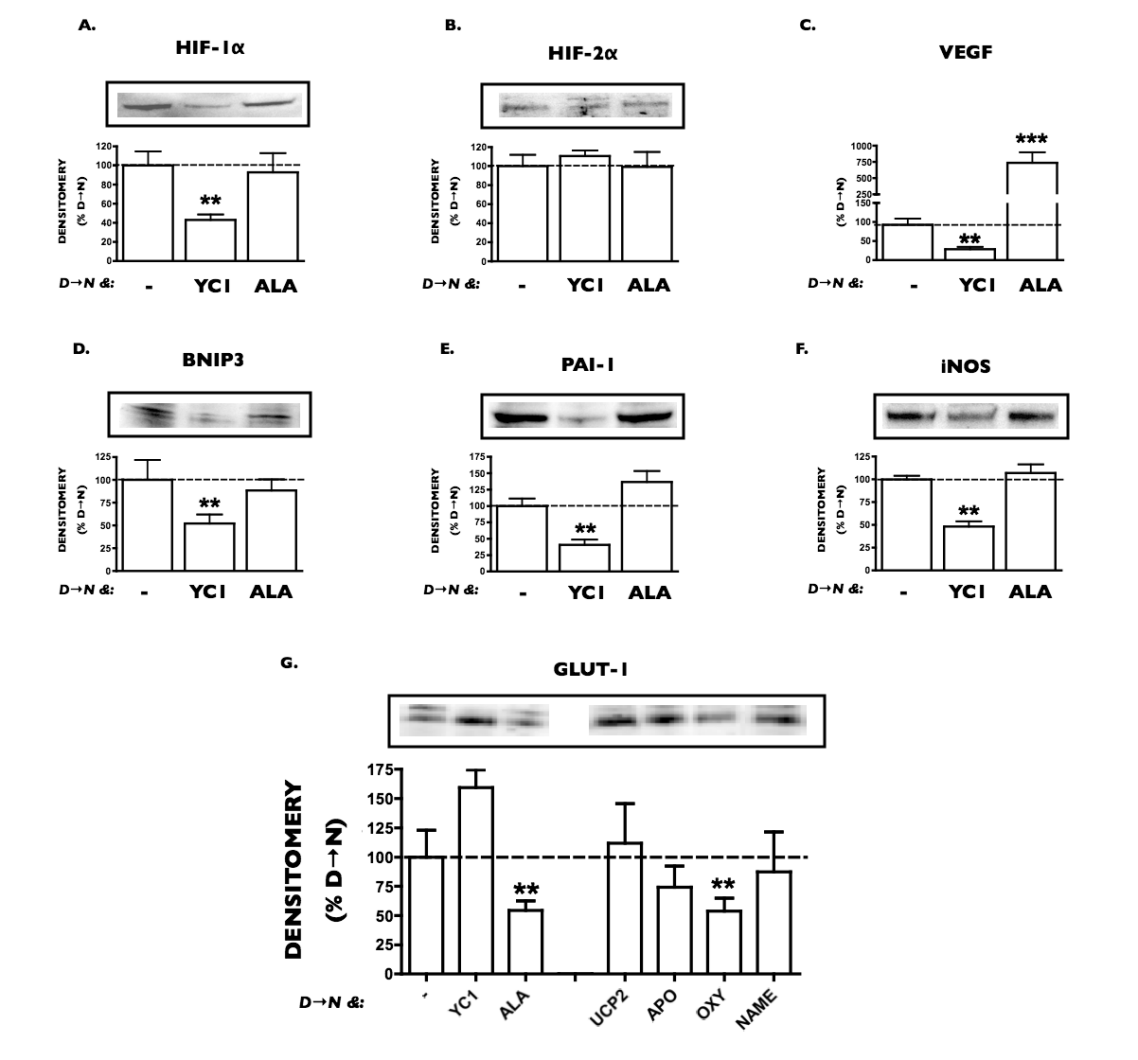
**Effect of various inhibitors and anti-oxidants on "memory" for HIF-1α, HIF-2α, Glut-1, BNIP3, PAI-1, iNOS and VEGF expression in human endothelial cells**. HUVECs were exposed to two weeks of mild decreased oxygen followed by one weeks of normoxia (**D**→**N**) in the presence or absence of 5 μM of the HIF inhibitor YC-1, or 62.5 μM α-lipoic acid (ALA), or 25–100 PFU/cell UCP2 adenovirus, or 10 μM apocynin (APO), or 10 μM oxypurinol (OXY) or to 1 mM L-NAME. Western blots were performed against HIF-1α (A), HIF-2α (B), BNIP3 (D), PAI-1 (E), iNOS (F) and GLUT-1 (G) on cellular lysates and ELISA was performed against VEGF (C) on cellular supernatants. Graphs for each Western blot indicate the densitometric analysis relative to the respective "memory" (**D**→**N**) protein expression (dashed line). Data in graphs represent the mean +/- S.E.M. from three separate experiments with a total sample size of 4–6 and with representative gels above each graph. Double asterisk, *P *< 0.01; triple asterisk, *P *< 0.001 as compared to cells in "memory" (**D**→**N**) treatment.

It has been suggested that a major regulator of HIF signaling in response to mild decreased oxygen is not the oxygen levels themselves, but rather a cellular buildup of reactive species secondary to decreased oxygen [55]. To address this, HIF and reactive species inhibitors were added during the last week of normoxia subsequent to two weeks of mild decreased oxygen in the HUVECs. The rationale for adding agents during the normalization period is to mimic a clinical situation with a patient with previous decreased oxygen exposure. To reduce HIF levels, short hairpin RNAs (hrRNAs) against HIF were developed. Adenoviral delivery of these siRNAs was found to reduce HIF-1α protein expression by 80–90% (Figure 6A). Unfortunately, all of these HIF shRNAs were found to be toxic to the cells (Figure 6B). In place of genetic manipulations, a well-characterized pharmacological agent, YC-1 [40], known to degrade the HIF-1α protein [56] was chosen and doses titrated to minimize cell death. It was found that 5 μM YC-1 resulted in approximately 60% reduction in the "memory" of induction of HIF-1α (Figure 7A) but not HIF-2α (Figure 7B) proteins with no significant toxicity to the cells (Figure 6B). YC-1 also resulted in a similar level of reduction in the "memory" of induction of the HIF target genes VEGF (Figure 7C), BNIP3 (Figure 7D), PAI-1 (Figure 7E) and iNOS (Figure 7F), but not GLUT-1 (Figure 7G) at the protein level.

### Reactive species mediate the persistent induction of GLUT-1 after oxygen normalization

To determine whether reactive species, a key non-hypoxic regulator of signaling [55], was involved in the "memory" of HIF signaling after oxygen normalization, the general antioxidant α-lipoic acid (ALA) [58] was added to the last week of normoxia. No change in the persistent induction of HIF-1α (Figure 7A), HIF-2α (Figure 7B), BNIP3 (Figure 7D), PAI-1 (Figure 7E), or iNOS (Figure 7F) proteins was observed with the addition of ALA. In contrast, a significant reduction in the persistent induction of GLUT-1 protein (Figure 7G) was observed with ALA. To examine the source of the reactive species which leading to the "memory" of GLUT-1 induction, the mitochondrial respiratory chain uncoupling protein UCP2; or the NAD(P)H oxidase inhibitor apocynin (APO); or oxypurinol (OXY), a xanthine oxidoreductase inhibitor; or L-NAME, an NOS inhibitor; were added during the last week of normalized oxygen. UCP, APO and L-NAME had little effect on the "memory" of GLUT-1 protein induction (Figure 7G) whereas OXY resulted in a partial but significant interruption of GLUT-1 induction.

### Persistent changes in HIF-1α ubiquitylation in response to chronic mild decreased oxygen

**Figure 8:**
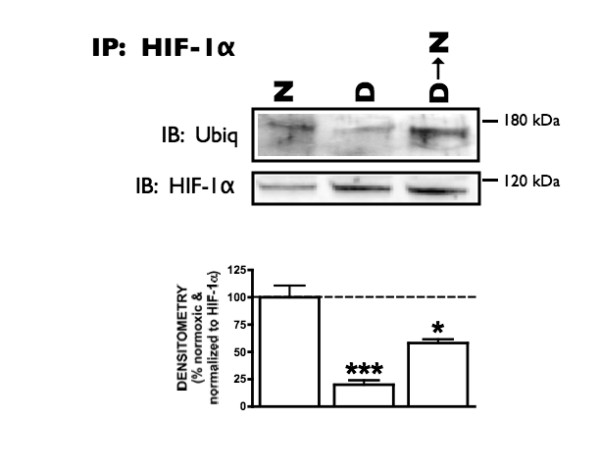
**Effect of the transition from mild hypoxia to normoxia on the ubiquitylation of HIF-α**. HUVECs were exposed to mild decreased oxygen as in Figure 1. Whole cell lysates were co-immunoprecipitated against HIF-1α and Western blot against ubiquitin (top panel) and HIF-1α (bottom panel) completed as in Materials and Methods. Densitometric analysis of ubiquitin expression in HUVECs graphed as a percentage of normoxic ubiquitin expression (dashed line). Data represent the mean +/- S.E.M. from three separate experiments with a total sample size of 4–6. Asterisk, *P *< 0.05; triple asterisk, *P *< 0.001 as compared to cells incubated in normoxia for three weeks.

To begin to explore the mechanistic basis behind the stabilization of the HIF-1α protein after oxygen normalization, the ubiquitylation of HIF-1α was examined in response to mild decreased oxygen. Using immunoprecipitation (IP) against HIF-1α and a Western blot against ubiquitin (Figure 8; top blot), it was found that chronic mild decreased oxygen (D) resulted in decreased ubiquitylation of HIF-1α while normalization of oxygen for one week after two weeks mild decreased oxygen (D>N) resulted in similar levels of ubiquitylation as cells exposed to normoxia (N). These ubiquitin blots were then re-probed with an HIF-1α antibody raised in another species (Figure 8; middle blots), and the densitometry of bands graphed as a ratio of ubiquitin:HIF-1α (Figure 8; bottom graph). It was found that chronic exposure to mild decreased oxygen, and to a lesser extent mild decreased oxygen followed by one week of oxygen normalization resulted in significantly decreased ubiquitin/HIF-1α ratios (Figure 8; bottom graph).

## Discussion

The present study demonstrates proof-of-principle that chronic exposure to mild decreased oxygen is capable of stimulating HIF signaling and that this induction of signaling persists one week after oxygen levels are normalized. We found this "memory" of HIF signaling was not cell type-specific, being observed in two different endothelial cell types as well as in aortic SMCs. We found that degrading the HIF-1α protein using the chemical YC-1 was capable of interrupting the "memory" of HIF-1α, VEGF, BNIP3, PAI-1 and iNOS induction and that decreasing cellular reactive species using a general antioxidant or an inhibitor of xanthine oxidoreductase was capable of interrupting the "memory" of GLUT-1 induction. Finally, we found that levels of ubiquitylated HIF-1α were reduced in response to chronic mild decreased oxygen and only partially recovered after oxygen levels were normalized. As a result of these findings, we speculate that HIF signaling may be contributing to the pathogenesis of endothelial dysfunction and could be further explored be a therapeutic target in addition to oxygen normalization.

The 15% O_2 _used in these studies is likely the mildest decrease in oxygen resulting in an induction of HIF signaling. This finding suggests that even such "innocuous" decreases in oxygen levels can trigger a hypoxic-like response in the endothelium. This could be a double-edged sword in terms of propagating endothelial dysfunction. Although purely speculative at this point, the persistent increase in GLUT-1 after oxygen normalization could result in increased glucose transport into the endothelium, which could exacerbate endothelial dysfunction in diabetic patients. The persistent induction of iNOS after oxygen normalization may increase NO available for vasodilation, but more likely the excess NO would combine with reactive oxygen species to form reactive nitrogen species, and again could worsen endothelial dysfunction [59]. The "memory" of VEGF induction after oxygen normalization could also be a double-edged sword, with the appropriate amount of VEGF facilitating neovascularization and an excess of VEGF leading to ill-formed vessels [60] and to increased vascular permeability and leukocyte infiltration [61]. Finally, high levels of PAI-1 have been found to be associated with cardiovascular disease [62], and BNIP3 has been found to be a mediator of myocardial death after infarction [63], again allowing one to surmise that the induction of these two HIF target genes could affect endothelial dysfunction.

In this work, both HIF α subunits were found to respond similarly to chronic mild decreased oxygen exposure, however, a "memory" of HIF-1α, but not HIF-2α was observed after one week of normalized oxygen levels following two weeks of mild decreased oxygen in both the microvascular (Figure 3) and umbilical vein (Figure 5) endothelial cells. In the aortic smooth muscle cells, however, a "memory" of HIF-2α protein induction was observed (Figure 5). This suggests, as others have implied, a differential regulation of these two HIF α subunits in response to certain stressors and in specific cell types [64,63,66]. This also supports the predominance of HIF-1α as compared to HIF-2α in some systems for regulating gene expression in response to decreased oxygen [67,66,62,64,70]. Although not examined in these studies, a decrease the endogenous HIF inhibitor HIF-3α [18] could also explain the cellular "memory" of HIF induction and ongoing studies in our laboratory are directed toward understanding the role of this protein in response to chronic mild decreased oxygen.

The "memory" of GLUT-1 induction after oxygen normalization was found to be mediated at least in part by reactive species, with an inhibitor of reactive species production through xanthine oxidoreductase resulting in a significant interruption of GLUT-1 "memory" (Figure 7G). This finding is supported by the work of Kelley et al. [70], who found that moderate hypoxia (10% O_2_) resulted in the induction of xanthine oxidoreductase in endothelial cells. An alternative to explain the cellular "memory" HIF induction could be that the *re*-oxygenation itself is stimulating HIF, perhaps via an increase in reactive species. While the addition of reactive species inhibitors and scavengers during the oxygen normalization period (Figure 7G) appears to rule this out in all cases except perhaps GLUT-1, this would be another interesting area of exploration. The addition of reactive species donors or other compounds like pro-inflammatory cytokines which increase reactive species together with the measurement of reactive species markers like 3-nitrotyrosine [71] and esterified glutathione [20] could be used to further address these issues.

The canonical mechanism of HIF induction in response to hypoxia, however, is not through reactive species but rather through HIF prolyl hydroxylase (HPH), enzymes, which target HIF α subunits for ubiquitylation and ultimate degradation by the proteasome and which are rendered inactive in response to low oxygen [72]. Based on many previous reports, it seemed improbable that the mild decreased oxygen (15% O_2_) used in this study is low enough to act through this mechanism [22, 72, 73]. Our results with ubiquitylated HIF-1α (Figure 8), however, suggest that HIF-1α ubiquitylation is reduced in response to chronic mild decreased oxygen and that ubiquitylation only partially recovers one week after oxygen levels were normalized. Ongoing work in our laboratory is seeking to examine whether the modification of HIF-1α by HPHs or whether the ubiquitylation of hydroxylated HIF-1α is the target for HIF stabilization in response to chronic mild decreased oxygen and upon oxygen normalization.

## Conclusion

In summary, our results provide a proof-of-principle that hypoxic signaling through HIF is induced in response to chronic mild decreased oxygen and that this induction persists long after oxygen levels are normalized. This work implies that the HIF system could be a relevant therapeutic target in the treatment of endothelial dysfunction. This work also upholds and extends a possible role of reactive species in the formation of endothelial dysfunction and bolsters the use of antioxidants and xanthine oxidoreductase inhibitors in the treatment of vascular disease in addition to oxygen normalization.

## Competing interests

The authors declare that they have no competing interests.

## Authors' contributions

CDK participated in the design of the studies, the experiments for figures 1–2, interpretation of data, statistical analysis, produced a first draft of the manuscript with JET, and was involved in subsequent drafts of the manuscript.

JET participated in the design of the studies, the experiments for figures 3, 4, 5, interpretation of data, statistical analysis, produced a first draft of the manuscript with CDK, and was involved in subsequent drafts of the manuscript.

SSS participated in the design of the studies, cell culture experiments, statistical analysis, and drafting of the manuscript.

AC, together with MAI, came up with the concept for a cellular "memory"of hypoxic stress signaling, designed the experiments, and was participated in drafting of the manuscript.

DEG participated in the cell culture studies and drafting of the manuscript.

LAW participated in the cell culture studies and drafting of the manuscript.

MAI, together with AC, came up with the concept of the cellular "memory"of hypoxic stress signaling, designed the studies, was intimately involved in data interpretation, statistical analysis, and in all phases of writing this manuscript.

All authors read and approved the final manuscript.

## Pre-publication history

The pre-publication history for this paper can be accessed here:
